# Internal Extractive Electrospray Ionization Mass Spectrometry for Quantitative Determination of Fluoroquinolones Captured by Magnetic Molecularly Imprinted Polymers from Raw Milk

**DOI:** 10.1038/s41598-017-15202-1

**Published:** 2017-11-07

**Authors:** Hua Zhang, Wei Kou, Aisha Bibi, Qiong Jia, Rui Su, Huanwen Chen, Keke Huang

**Affiliations:** 1 State Key Laboratory of Inorganic Synthesis and Preparative Chemistry, College of Chemistry, Jilin University, Changchun, 130012 P.R. China; 2 Changchun University of Chinese Medicine, Changchun, 130117 P.R. China; 3 Jiangxi Key Laboratory for Mass Spectrometry and Instrumentation, East China University of Technology, Nanchang, 330013 P.R. China

## Abstract

Antibiotics contamination in food products is of increasing concern due to their potential threat on human health. Herein solid-phase extraction based on magnetic molecularly imprinted polymers coupled with internal extractive electrospray ionization mass spectrometry (MMIPs-SPE-iEESI-MS) was designed for the quantitative analysis of trace fluoroquinolones (FQs) in raw milk samples. FQs in the raw milk sample (2 mL) were selectively captured by the easily-lab-made magnetic molecularly imprinted polymers (MMIPs), and then directly eluted by 100 *µ*L electrospraying solvent biased with +3.0 kV to produce protonated FQs ions for mass spectrometric characterization. Satisfactory analytical performance was obtained in the quantitative analysis of three kinds of FQs (*i*.*e*., norfloxacin, enoxacin, and fleroxacin). For all the samples tested, the established method showed a low limit of detection (LOD ≤ 0.03 *µ*g L^−1^) and a high analysis speed (≤4 min per sample). The analytical performance for real sample analysis was validated by a nationally standardized protocol using LC-MS, resulting in acceptable relative error values from −5.8% to +6.9% for 6 tested samples. Our results demonstrate that MMIPs-SPE-iEESI-MS is a new strategy for the quantitative analysis of FQs in complex biological mixtures such as raw milk, showing promising applications in food safety control and biofluid sample analysis.

## Introduction

Antibiotics have been widely used for decades to effectively treat a variety of bacterial infections, and great contributions have been made in human health protection. Unfortunately, because of worldwide overuse and misuse of antibiotics in planting and breeding production process^1–3^, bacteria are often becoming strongly resistant to hospital treatment^4,5^. Thus the antibiotics contamination in food products is of increasing concern due to their hazardous effects on human health and ecosystem^1,6–9^, which include but not limited to the infections caused from antibiotic-resistant bacteria and possible carcinogenicity^10–12^. Among series of antibiotics, fluoroquinolones (FQs) are one kind of broad-spectrum antibiotics, which are ubiquitously used in human health care and veterinary applications^13^.

Conventional analytical methods including microbiological methods^14^, electrochemical method^15^, fluorospectrophotometry^16^, high performance thin layer chromatography (HPTLC)^17^, high performance liquid chromatography with ultraviolet detector (HPLC-UV)^18^, high performance liquid chromatography mass spectrometry (HPLC-MS)^19^ and enzyme immunoassay^20^ have been applied to the detection of FQs in environment water, foodstuffs, and biofluid samples, *etc*. Although spectroscopy detection methods (*e*.*g*., ultraviolet detector) have been widely used in the determination of FQs benefited by the chromophore or fluorophore groups in the FQs molecule^21^, its limited sensitivity may be a challenge in specific application. Moreover, tedious sample pretreatments (*e*.*g*., centrifugation, diluting, and multistep chemical extraction, *etc*.) for the matrix clean-up are routinely required, which prevents the high-throughput analysis of FQs in practical samples. Thus, there is an urgent demand for the development of highly efficient analytical methods of sensitive and selective identification or quantification of FQs in samples with complex matrices.

Recently, ambient mass spectrometry (AMS) allows the direct analysis of complex samples with high speed, high selectivity, and high sensitivity^22–24^. Charged droplet generated by electro-spray or sonic spray is a common ionization reagent, which is widely used in various ambient ionization technologies such as desorption electrospray ionization (DESI)^25^, probe electrospray ionization (PESI)^26^, extractive electrospray ionization (EESI)^27^, laser ablation electrospray ionization (LAESI)^28^, and easy ambient sonic spray ionization (EASI)^29^, *etc*. Benefited by the high ionization energy, the primary ions generated by electric field (electron/plasma) have been employed in many ambient ionization technologies including direct analysis in real time (DART)^30^, low temperature plasma (LTP)^31^, microwave plasma torch (MPT)^32^, plasma assisted laser desorption ionization (PALDI)^33^, dielectric barrier discharge ionization (DBDI)^34^, desorption atmospheric pressure chemical ionization (DAPCI)^35^, *etc*., which are of unique advantages for the preparation of specific analytes ions from raw samples. Great convenience has been provided by these versatile ambient ionization technologies owing to the direct sampling or ionization of raw samples. To date, efforts are still devoting to improve the analytical performance of AMS facing highly complex matrices. In recent years, fast and facile sample pretreatment methods (*e*.*g*., solid-phase microextraction (SPME)^36,37^, magnetic solid-phase extraction (MSPE)^38^, thin-layer chromatography^39^, solid phase mesh enhanced sorption from headspace (SPMESH)^40^, *etc*.) combined with AMS has been developed for direct analysis of trace target analytes in various highly complex samples (*e*.*g*., biological, environmental, food, forensic samples, or even individual small organism), which greatly improved the sensitivity and selectivity of AMS.

Given raw milk as a typical example of extremely complex matrix, a facile method of solid-phase extraction based on magnetic molecularly imprinted polymers combined with internal extractive electrospray ionization^41–43^ mass spectrometry (MMIPs-SPE-iEESI-MS) was designed for the quantitative analysis of FQs in raw milk samples. FQs in the raw milk samples were selectively captured by the MMIPs for subsequent iEESI-MS interrogation. Overall, the established method showed a high sensitivity in the determination of three kinds of FQs (norfloxacin, enoxacin, and fleroxacin) in raw milk samples. Our results demonstrate that the established MMIPs-SPE-iEESI-MS is a powerful method for the quantitative analysis of FQs in raw milk samples, providing potential application value in other biofluid sample analysis.

## Results

### MMIPs-SPE-iEESI-MS Analysis of FQs in raw milk samples

**Figure 1:**
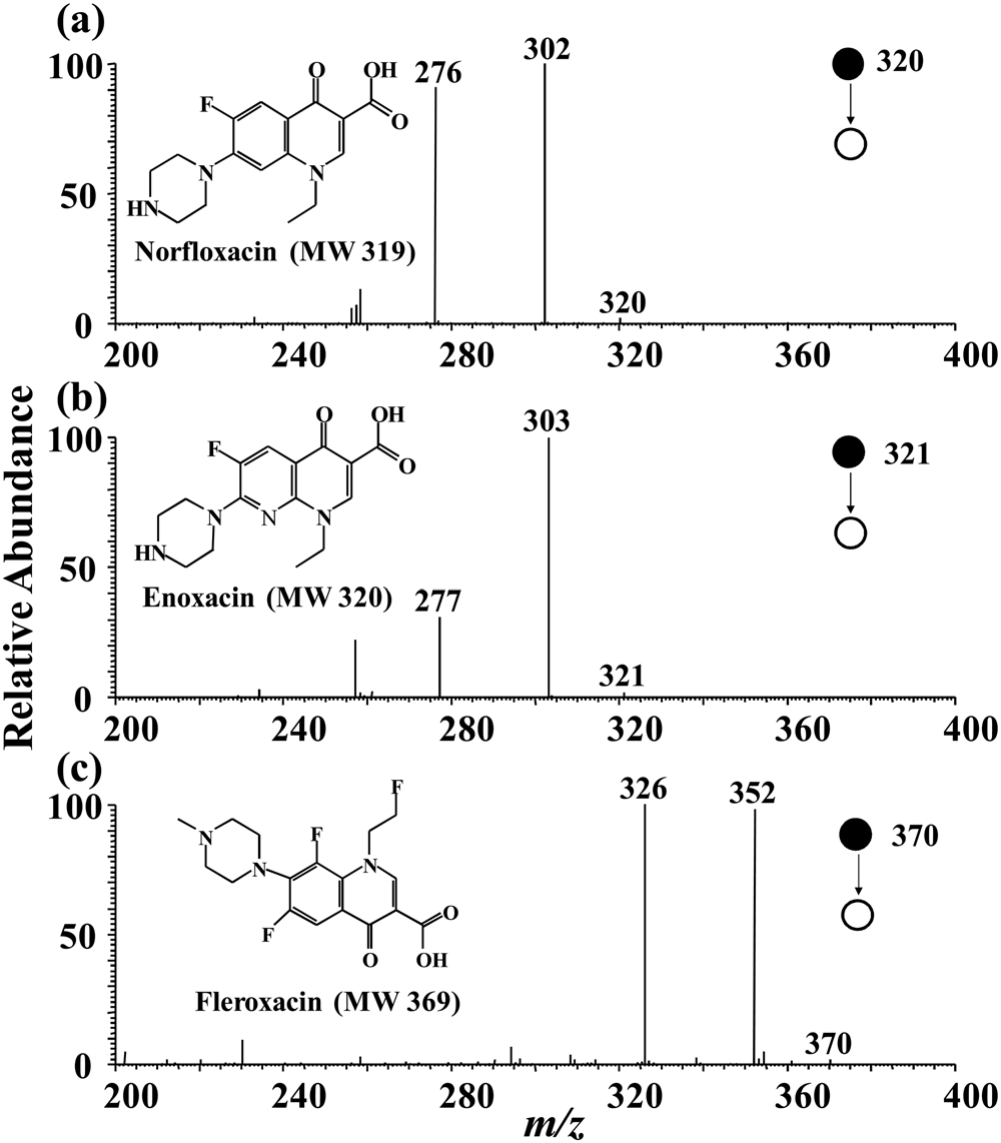
Typical MMIPs-SPE-iEESI-MS/MS spectra of FQs spiked in raw milk samples. (**a**) Norfloxacin, (**b**) enoxacin, and (**c**) fleroxacin.

To exclude false positive result for the analysis of FQs in milk samples, collision-induced dissociation (CID) experiments were performed for all the suspected FQs protonated molecule ions of *m/z* 320, *m/z* 321, and *m/z* 370. Figure 1 shows the MS/MS spectra of precursor ions of *m/z* 320, *m/z* 321, and *m/z* 370 collected from raw milk samples with authentic FQs (10 *μ*g L^−1^). Fragment ions of (*m/z* 302, *m/z* 276), (*m/z* 303, *m/z* 277), and (*m/z* 352, *m/z* 326) were yielded by the precursor ions of *m/z* 320, *m/z* 321, and *m/z* 370, respectively, which were consistent with characteristic fragment ions produced by protonated molecule ions of [norfloxacin +H]^+^ (*m/z* 320), [enoxacin +H]^+^ (*m/z* 321), and [fleroxacin +H]^+^ (*m/z* 370) according to previous literatures^44,45^. All the protonated molecule ions of [norfloxacin +H]^+^, [enoxacin +H]^+^, and [fleroxacin +H]^+^ were easily to occur neutral loss of H_2_O and CO_2_ under the CID conditions. The loss of CO_2_ (−44) got characteristic fragment ions of *m/z* 276, *m/z* 277, and *m/z* 326 by precursor ions of norfloxacin, enoxacin, and fleroxacin, respectively. These CO_2_ (−44) lost fragment ions should provide higher significance for the identity check of these three FQs. Thus, the signal intensities of fragment ions of *m/z* 276, *m/z* 277, and *m/z* 326 were selected as analytical response to establish the quantitative methods for norfloxacin, enoxacin, and fleroxacin, respectively. As a result, the FQs in the raw milk samples were successfully detected using MMIPs-SPE-iEESI-MS.

### Optimization of MMIPs-SPE-iEESI

For better performance during MMIPs-SPE-iEESI-MS analysis, analytical parameters including sorbent amount, composition, volume of extraction solvent, and the flow rate of extraction were optimized using FQs spiked raw milk as samples. The concentration of each FQs (*i*.*e*., norfloxacin, enoxacin, and fleroxacin) was set at 10 *μ*g L^−1^ in all the milk samples.

**Figure 2:**
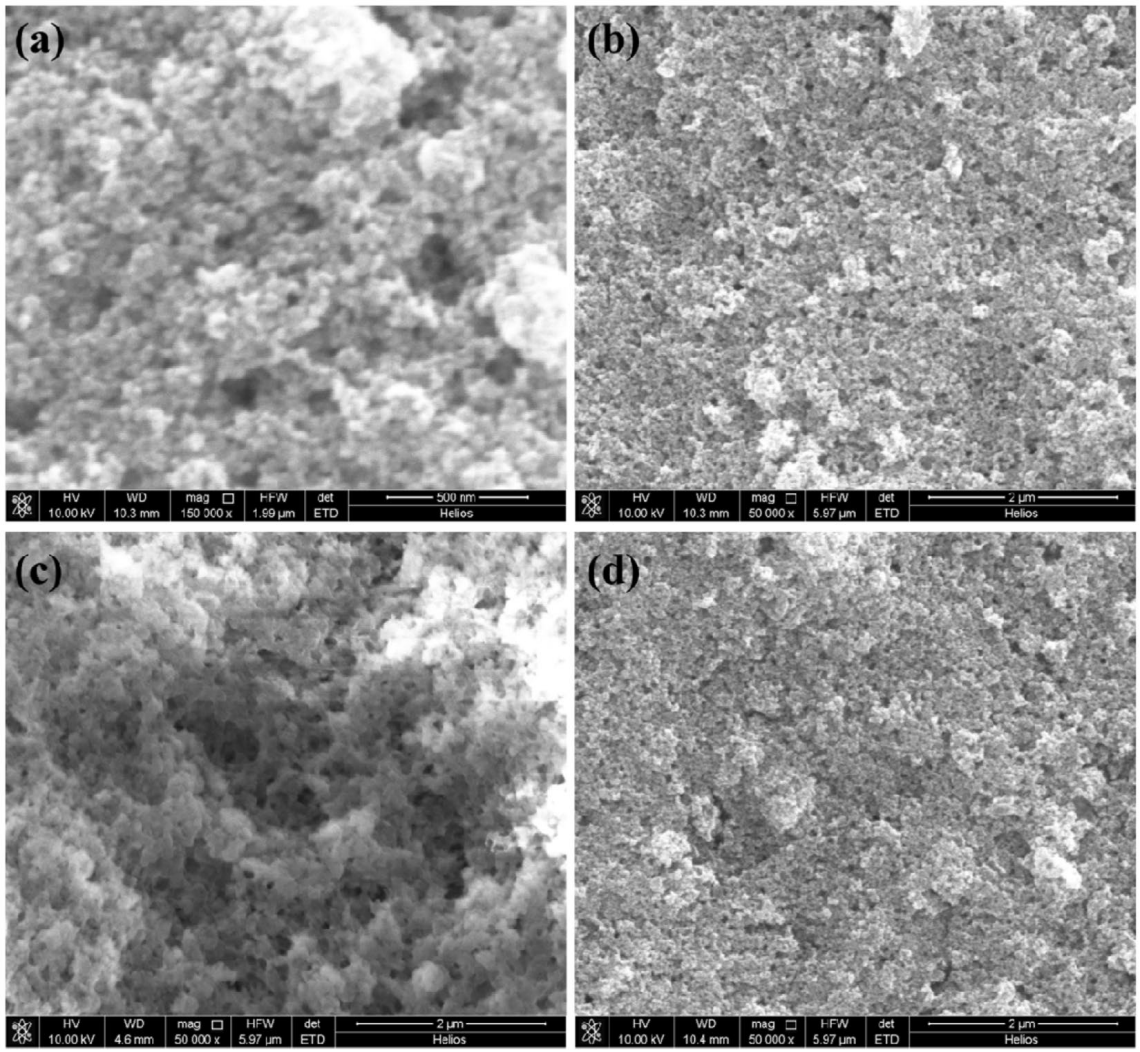
SEM images of the materials: (**a**, **b**) Fe_3_O_4_ magnetite nanoparticles (MNPs), (**c**) molecularly imprinted polymers (MIPs), (**d**) magnetic molecularly imprinted polymers (MMIPs).

**Figure 3:**
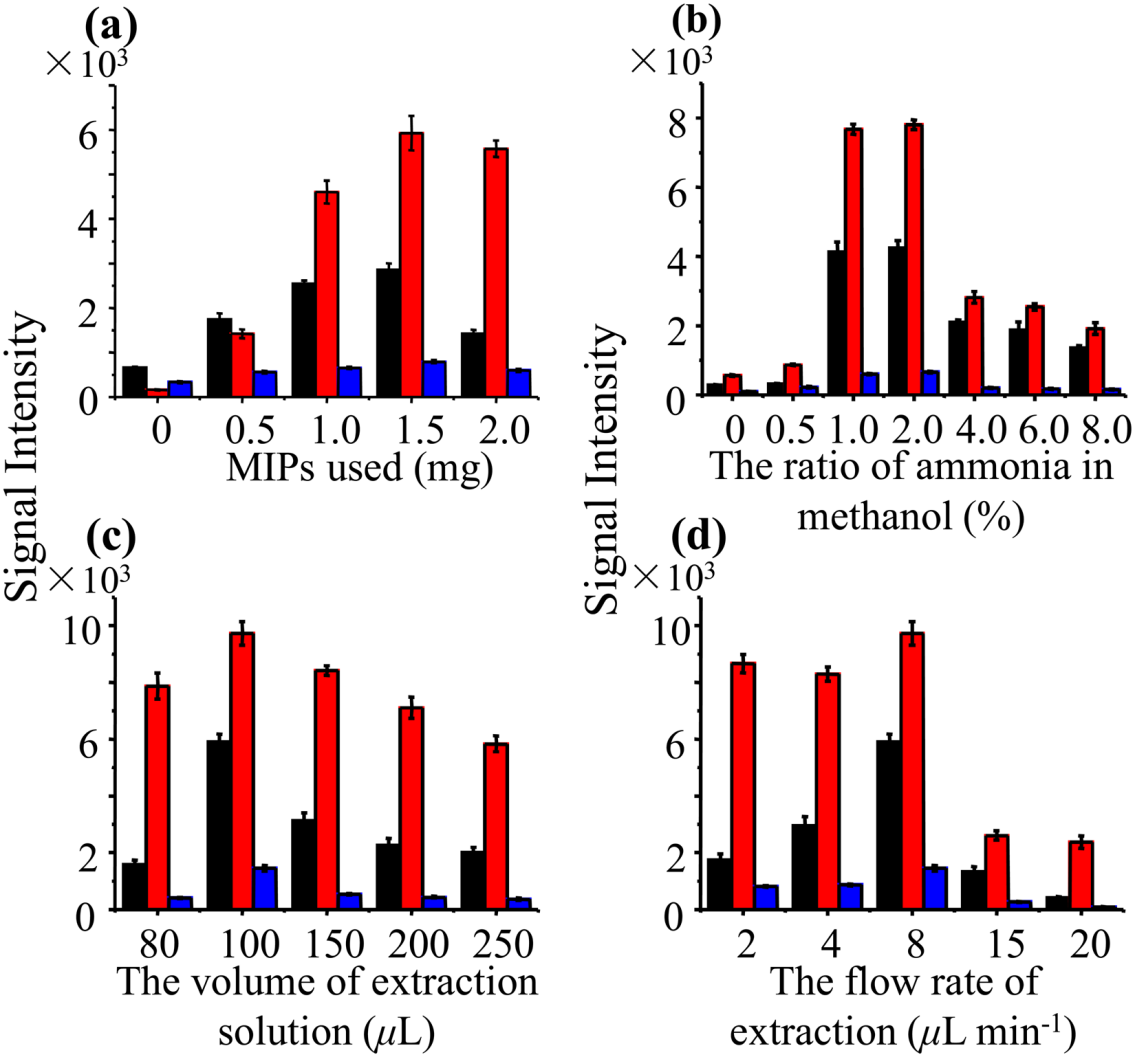
Optimization of the MMIPs-SPE-iEESI experimental conditions. (**a**) Amount of MIPs, (**b**) composition of extraction solution, (**c**) volume of extraction solution, and (**d**) flow rate of extraction. The black, red, and blue columns present the signal intensities of norfloxacin (*m/z* 276), enoxacin (*m/z* 277), and fleroxacin (*m/z* 326), respectively.

MMIPs material was simply fabricated by co-mixing of Fe_3_O_4_ magnetic nanoparticles (MNPs) and a commercial molecularly imprinted polymers (MIPs) products in methanol. As shown in the SEM image of MMIPs material (Fig. 2), the MNPs were coated on the surface of the MIPs after the co-mixing preparation. Additional elemental analysis of the MMIPs, Fe_3_O_4_ MNPs, and MIPs also imply the assembly of Fe_3_O_4_ MNPs and MIPs (Supplementary Fig. S1). A comparison experiment of the Fe_3_O_4_ MNP material (without MIPs) and the MMIP material (with MIPs) was carried out. As expected, the target FQs signals were remarkably increased when using MMIP material (Supplementary Fig. S2). To achieve high adsorption performance for the FQs, different amounts of MIPs material (*i*.*e*., 0, 0.5, 1.5, and 2.0 mg) were experimentally investigated for FQs adsorption, while the amount of MNPs was kept at 2.0 mg. The signal intensities of the three FQs notably increased with the increase of MIPs amount from 0 to 1.5 mg, and showed a decreasing trend when the MIPs amount increased to 2.0 mg (Fig. 3a). As a result, 1.5 mg MIPs and 2.0 mg MNPs were used for the preparation of MMIPs material. As shown in the SEM image of MMIPs material (Fig. 2), the MNPs were coated on the surface of the MIPs material. Considering the extraction solution was acted as both the elution solution for FQs desorption and the solution for electrospray, the extraction solution was also investigated. Methanol containing with different proportion of ammonia 0%, 0.5%, 1.0%, 2.0%, 4.0%, 6.0%, and 8.0% (w/w) were applied for the MMIPs-SPE-iEESI-MS analysis. As a result, 2.0% ammonia in methanol (w/w) was the optimal extraction solution (Fig. 3b). The increased ammonia proportion in methanol should be helpful for the desorption of FQs, while excessively high concentration of ammonia (*e*.*g*., 8.0%, w/w) may suppress the ionization efficiency of FQs. Moreover, the volume of the extraction solution for the elution of FQs from the MMIPs material and the flow rate of the solution were also optimized to achieve better elution and ionization efficiency. Higher FQs signal intensity was obtained under a volume of 100 *μ*L and a flow rate at 8 *μ*L min^−1^ (Fig. 3c and d). Finally, optimized conditions showed satisfactory performance for the determination of three kinds of FQs in raw milk samples.

### Quantitative analysis of FQs in milk samples using MMIPs-SPE-iEESI-MS

**Figure 4:**
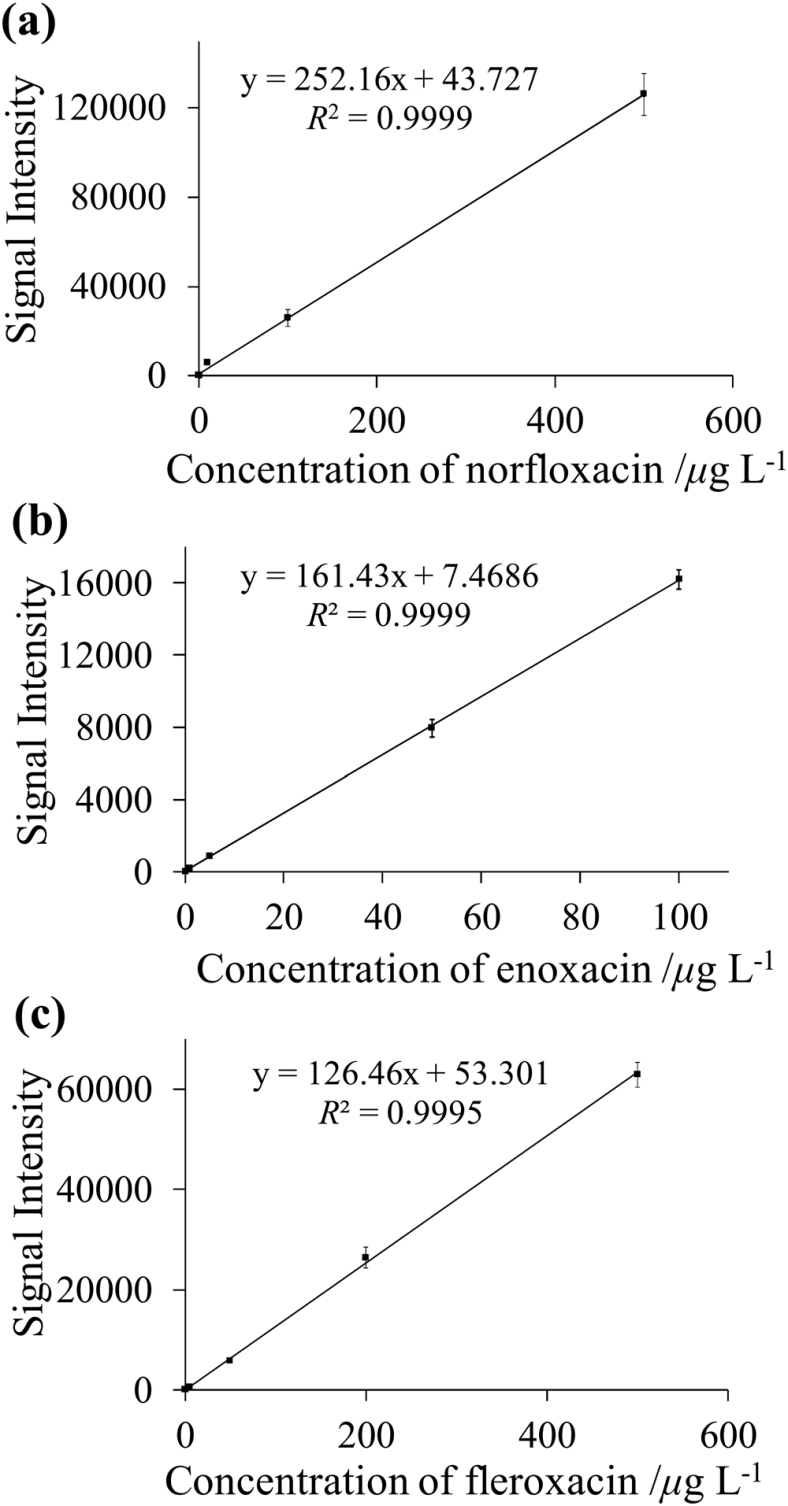
The intensity levels of the characteristic fragments of FQs against the concentrations (*µ*g L^−1^) of FQs in milk sample solution. (**a**) Norfloxacin, (**b**) enoxacin, and (**c**) fleroxacin.

Three kinds of FQs standard solutions (*i*.*e*., norfloxacin, enoxacin, and fleroxacin, respectively) were spiked in blank raw milk samples (2 mL) to make a series of working solutions containing 0.1–500.0 *μ*g L^−1^ of FQs for MMIPs-SPE-iEESI-MS/MS analysis. In the case of norfloxacin, the signal intensity of *m/z* 276 was linearly responded with norfloxacin concentrations over the range of 0.1–500.0 *μ*g L^−1^ (*R* ^2^ = 0.9999) (Fig. 4a). The LOD of norfloxacin defined by a signal-to-noise ratio (S/N) of 3 was estimated to be 0.019 *μ*g L^−1^. The relative standard deviations (RSDs) of six replicates for the norfloxacin concentrations ranging from 0.1–500.0 *μ*g L^−1^ were less than 8.7% (detailed in Supplementary Table S1). For the quantitative analysis of enoxacin and fleroxacin, the linear responding ranges and relative standard deviation values (n = 6) were 0.1–100.0 *µ*g L^−1^ (*R* ^2^ = 0.9999) and less than 7.5% for enoxacin (Fig. 4b and detailed in Supplementary Table S2), and 0.1–500.0 *µ*g L^−1^ (*R* ^2^ = 0.9995) and less than 8.4% for fleroxacin (Fig. 4c and detailed in Supplementary Table S3), respectively. The LODs defined by a signal-to-noise ratio (S/N) of 3 were estimated to be 0.022 *μ*g L^−1^ for enoxacin and 0.024 *μ*g L^−1^ for fleroxacin (Table 1), respectively. A short time estimated less than 4 min (exclude the time of MMIPs preparation) was taken for each measurement. Recoveries of all the three FQs from raw milk samples were also estimated by analyzing spiked samples. Acceptable recoveries from 82.5% to 110.0% were obtained for all the samples, and RSDs (n = 6) of all spiked samples were less than 9.4% (Table 1). Furthermore, intra/inter-day precision and accuracy of the method were carried out with the FQs spiked at three different concentrations in milk samples. The intra-day precision and accuracy were determined on the same day and consisted of six replicates at each of three concentration levels, and the inter-day precision and accuracy were carried out with a continuous fourteen days. The results obtained are shown in Table 2. The intra- and inter-day RSDs were less than 8.2% and 10.9%, respectively, while the intra- and inter-day recoveries ranging from 84.7 to 104.8% and from 85.9 to 105.6% were obtained, respectively.

**Table 1:** Recoveries obtained for MMIPs-SPE-iEESI-MS/MS analysis of three kinds of FQs spiked in milk samples.

Analytes	Linear range (*μ*g L^−1^)	*R* ^2^	LOD (*μ*g L^−1^)	Spiked FQs in milk sample (*μ*g L^−1^)	Recovery (%, n = 6)	RSD of measured concentrations (%, n = 6)
Norfloxacin	0.1–500.0	0.9999	0.019	0.3 ^a^	86.9	7.2
				5 ^a^	99.1	9.1
				50 ^a^	105.1	8.9
				300 ^a^	102.4	7.9
				400 ^a^	110.0	5.1
Enoxacin	0.1–100.0	0.9999	0.022	0.3 ^b^	93.6	9.4
				10 ^b^	91.5	7.0
				20 ^b^	94.2	8.7
				70	82.5	3.9
				80 ^b^	96.1	6.3
Fleroxacin	0.1–500.0	0.9995	0.024	0.3 ^c^	104.3	9.4
				10 ^c^	92.4	7.8
				100 ^c^	90.9	8.3
				300 ^c^	92.6	7.1
				400 ^c^	96.7	5.4

^a ^Blank milk samples were spiked with a series of concentration (*µ*g L^−1^) of norfloxacin. ^b ^Blank milk samples were spiked with a series of concentration (*µ*g L^−1^) of enoxacin. ^c ^Blank milk samples were spiked with a series of concentration (*µ*g L^−1^) of fleroxacin.

**Table 2:** Method precisions and accuracies at three concentrations for the determination of FQs from raw milk sample.

Analytes	Spiked FQs in milk samples (μg L^−1^)	Detected results			
		Intra-day precision (RSD, %, n = 6)	Intra-day accuracy (recovery, %, n = 6)	Inter-day precision (RSD, %, n = 14)	Inter-day accuracy (recovery, %, n = 14)
Norfloxacin	5.00 ^a^	7.1	104.8	8.7	104.0
	50.00 ^a^	5.4	100.8	6.7	104.8
	300.00 ^a^	7.4	101.4	9.1	96.6
Enoxacin	10.00 ^a^	2.9	88.7	5.5	91.1
	20.00 ^a^	6.0	101.6	7.7	101.7
	70.00 ^a^	7.6	91.2	9.1	87.6
Fleroxacin	10.00 ^a^	3.9	101.2	10.9	105.6
	100.00 ^a^	8.2	102.4	9.3	91.3
	300.00 ^a^	2.8	84.7	6.9	85.9

^a^Blank milk samples were spiked with a series of concentration (*µ*g L^−1^) of norfloxacin, enoxacin, fleroxacin, respectively.

### Method validation

Validation of the analytical results of MMIPs-SPE-iEESI-MS for detection of trace FQs in milk samples were performed using the conventional off-line LC-MS/MS method according to a standard operation procedure recommended on *National Standard of China* (*GB*/*T 22985*-*2008*) (detailed in Supplementary). As summarized in Table 3, the MMIPs-SPE-iEESI-MS results were all in good agreement with those obtained by LC-MS/MS. The good recovery rates (94.2–106.9%) and relatively low relative errors (−5.8% to +6.9%) confirmed that the MMIPs-SPE-iEESI-MS perfectly meet the requirement for the quantitative determination of FQs in raw milk samples.

**Table 3:** Validation of MMIPs-SPE-iEESI-MS/MS method with the LC-MS/MS method of *National Standard of China*, *GB/T 22985-2008*.

Analytes	Spiked FQs in milk samples (μg L^−1^)	Detected results					
		LC-MS ^a^			MMIPs-SPE-iEESI-MS		
		Detected value (*μ*g L^−1^, n = 3)	Recovery (%, n = 3)	RE ^b^ (%)	Detected value (*μ*g L^−1^, n = 3)	Recovery (%, n = 3)	RE ^b^ (%)
Norfloxacin	50.00 ^c^	48.60 ± 0.70	97.2	−2.8	49.40 ± 3.57	98.8	−1.2
	300.00 ^c^	306.00 ± 3.00	102.0	+2.0	320.65 ± 19.83	106.9	+6.9
Enoxacin	10.00 ^c^	9.10 ± 0.68	91.0	−9.0	9.43 ± 0.68	94.3	−5.7
	20.00 ^c^	20.13 ± 0.45	100.6	+0.7	19.47 ± 0.68	97.4	−2.7
Fleroxacin	100.00 ^c^	94.17 ± 1.04	94.1	−5.8	94.21 ± 2.63	94.2	−5.8
	300.00 ^c^	304.83 ± 0.29	101.6	+1.6	293.48 ± 13.18	97.8	−2.2

^a ^LC-MS method following the experimental protocol on *National Standard of China*, *GB/T 22985-2008*. ^b ^RE, relative error, calculated by treating the concentration of spiked FQs as practical real value. ^c ^Blank milk samples were spiked with a series of concentration (*µ*g L^−1^) of norfloxacin, enoxacin, fleroxacin, respectively.

## Discussion

In the optimization of the MMIPs amounts, the signal intensities of three kinds of FQs were increased with the increase of MIPs amounts from 0 to 1.5 mg, indicating that more FQs molecules in the complex milk sample were captured by the MMIPs material, which is consistent with higher ratio of MIPs. Interestingly, the signal intensities were decreased by using 2.0 mg MIPs. The preparation of MMIPs by co-mixing method was interpreted as “aggregate-wrap” process, *i*.*e*., the MIPs were likely to be wrapped by MNPs and aggregated to form a magnetic composite^46,47^. In this respect, sufficient MNPs were necessary to ensure all the MIPs material could be magnetic coated for the milk matrix separation. As the amount of MNPs was fixed at 2.0 mg, the magnetism of the MMIPs particles was decreased when more mass of MIPs (*e*.*g*., 2.0 mg) added for the assembly, resulting part of the MMIPs material loss during the solid-liquid separation. Also, a higher mass of MMIPs material might cause serious aggregation effect, which hindered the elution of FQs with a fixed volume of elution solution. Thus, a lower FQs signal was obtained. Of course, more detailed material properties of the MMIPs and the spontaneous assembling mechanism of MNPs and MIPs will subject to our further studies.

Matrix effects from highly complex samples are a great challenge on the quantitative analysis of AMS because of serious ion suppression. To achieve highly sensitive and selective determination of trace analytes in complex samples, coupling simple, rapid and sensitive sample pretreatment methods to AMS is a promising strategy to improve the performance of AMS^48–51^. Raw milk is a typical extremely complex sample which cannot introduce to MS analysis directly. To address this problem, a facile method of solid-phase extraction based on magnetic molecularly imprinted polymers (MMIPs) combined with iEESI-MS was designed for the quantitative analysis of FQs in raw milk samples. The FQs molecules in the milk were selectively adsorbed by MMIPs and the MMIPs (together with the adsorbed FQs) was separated from the milk matrix. Thus, the majority of the milk matrix was cleaned up. Additionally, to avoid the milk residues interference, the separated MMIPs material was washed three times using 1 mL deionized water, acetonitrile, and 15% acetonitrile in deionized water (v/v), respectively. As a result, the matrix of the milk was largely cleared. The target analytes are sequestered by MMIPs and directly analyzed by iEESI-MS. Due to the highly selective extraction of MMIPs, ionic suppression is minimized; hence no chromatographic separation is necessary, which greatly increases analytical speed and sensitivity. Moreover, during the MS interrogation, CID experiments were carried for the suspected FQs ions, *i*.*e*., the FQs were identified based on their characteristic fragment ions, which practically avoid false positive result. Our results demonstrate that MMIPs-SPE-iEESI-MS enables direct quantification of sub-ppb level of FQs in raw milk samples without tedious sample pretreatments (*e*.*g*., centrifugation and chemical extraction). Furthermore, a comprehensive analytical performance comparison of the proposed MMIPs-SPE-iEESI-MS method with those of previous reported methods^46,52–56^ in the analysis of FQs is presented in Table 4. The data showed that the method established in this work was of higher speed and better sensitivity than those previously reported methods.

**Table 4:** Comparison of proposed MMIPs-SPE-iEESI-MS/MS method in this study with other methods for detection of FQs residues.

Techniques	Samples	Analytes ^a^	Time required (min)	Determination	LODs	Ref
MIMSPE	Milk	OFL, CIP, LOM	4.5	LC-UV	1.8–3.2 ng/mL	46
MISPE	Urine	CIP, etc.	>20	LC-FD	1.9–34 ng/mL	52
MIPMME	Milk	CIP, etc.	>20	LC-FD	0.4–1.6 ng/mL	53
MMF-SPME	Honey	FQs, etc.	>130	HPLC-MS/MS	1.0–2.8 ng/Kg	54
Electrochemicaly enhanced SPME (based on MIPs)	Urine, soil	OFL, etc.	75	LC-DAD	0.5–1.9 ng/mL	55
MSPE	Milk	SAs, etc.	>30	HPLC-MS/MS	0.02–0.13 ng/mL	56
MIMSPE	Milk	NOR, ENO, FLE	≤4	iEESI-MS/MS	0.019–0.024 ng/mL	This work

^a^Abbreviations: CIP, ciprofloxacin; ENO, enoxacin; FLE, fleroxacin; FQs, fluoroquinolones; LOM, lomefloxacin; NOR, norfloxacin; OFL, ofloxacin; SAs, sulfonamides.

Combination of MMIPs-SPE with iEESI-MS was benefited by the high performance of MMIPs material in the capture of FQs from milk (*i*.*e*., fast and easy sample matrix clean-up step), as well as the specially designed sample loading/ionization process of iEESI. Molecularly imprinted polymers (MIPs) are a class of material engineered to bind one target compound or a class of structurally related compounds with high selectivity^57,58^. Due to the highly selectivity of MIPs, ionic suppression during ESI could be minimized, *e*.*g*., Figueiredo *et al*. employed MIP-SPE in ESI-MS for analysis of drugs in human plasma^59^. Although merits such as no chromatographic separation and minimized ionic suppression were achieved in the combination of MIP-SPE-ESI^59^, but tedious and laborious sample pretreatments including liquid-liquid extraction (for proteins elimination), centrifugation, preconcentration, sample re-dissolution, *etc*. were still needed on the account of highly complex of the plasma sample. In this respect, ambient ionization technologies provide a unique strategy for direct sampling/ionization analytes from the sample with no/minimum sample pretreatments^22,60,61^. Undoubtedly, the combination of facile sample pretreatment strategies (*e*.*g*., SPE, SPME, *etc*.) and ambient ionization methods is of promising when facing highly complex samples such as plasma and milk samples^48–51^. iEESI belongs to the ambient ionization methods family, which has been developed as a direct and fast sampling and ionization method for mass spectrometric analysis of complex samples^41,62^. Combination of SPE method and iEESI is a promising strategy to improve the analytical performance of iEESI. In a previous study, coupling of magnetic solid-phase extraction with iEESI was developed to study 1-hydroxypyrene in undiluted human urine samples with the assistance of polypyrrole-coated Fe_3_O_4_ magnetite nanocomposites (Fe_3_O_4_@Ppy nanocomposites)^42^. Due the low polarity of the coated polypyrrole on the surface of Fe_3_O_4_ magnetite nanocomposites, chemicals of low polarity (*e*.*g*., 1-hydroxypyrene and 3-hydroxybenzeo[a]pyrene, *etc*.) were easily captured for subsequent iEESI-MS analysis. The selectivity of Fe_3_O_4_@Ppy nanocomposites is a notable drawback when treating chemicals with similar polarity^38,42,63^. To address this concern, highly selectivity and specificity could be introduced by MIPs material. Selectivity of MIPs is introduced during MIPs synthesis in which a template molecule, designed to mimic the analyte, guides the formation of specific cavities that are sterically and chemically complementary to the target analytes^64,65^. Strong retention is offered between a MIP phase and its target analyte(s) based on multiple interactions (*e*.*g*., Van der Waals, hydrogen bonding, ionic, hydrophobic) between the MIP cavity and analyte functional groups^64,65^. As a result, even trace FQs in the raw milk were captured and subsequently subject to iEESI-MS.

To conclude, combination of fast and easy-to-use sample pretreatment with mass spectrometry is a promising strategy for high throughput quantitative detection of trace analytes in highly complex samples. As a typical example of the analytical strategy, MMIPs-SPE-iEESI-MS was designed for the confidently quantitative analysis of FQs in raw milk samples. As a result, FQs in the raw milk sample were selectively enriched by the MMIPs and then directly eluted by the electrospraying solvent to produce protonated FQs ions for mass spectrometric interrogation. The LOD of ≤0.03 *µ*g L^−1^ and the high speed of 4 min for per sample were achieved. The analytical performance for real sample analysis was validated by a nationally standardized protocol using LC-MS, resulting in acceptable relative errors from −5.8% to +6.9% for 6 tested samples. Our results demonstrate that MMIPs-SPE-iEESI-MS is a facile method for the high throughput quantitative analysis of FQs in raw milk samples, which shows promising applications in food safety control and biofluid sample analysis.

## Methods

### Materials and chemicals

Water-compatible commercial molecularly imprinted polymers (MIPs) material, named SupelMIP^TM^ SPE-Fluoroquinolones, was purchased from Sigma–Aldrich (St.Louis, MO, USA). Fe_3_O_4_ magnetite nanocomposites (MNPs) were prepared according to previous studies^46,66^ (detailed in Supplementary). Fluoroquinolones (norfloxacin, enoxacin, and fleroxacin, purity 98%) were purchased from J&K Scientific Ltd. (Shanghai, China). The individual stock solutions of norfloxacin, enoxacin, and fleroxacin were prepared in methanol at a concentration of 0.1 mg mL^−1^ and stored at 4 °C before use. Both Methanol and acetonitrile were HPLC grade and purchased from Merck KGaA (Darmstadt, Germany). Ammonium hydroxide solution (suitability for use in UPLC/LC-MS, NH_3_, w/w 20%) was bought from CNW Technologies GmbH (Düsseldorf, Germany). Ethylene glycol, ethylene diamine, ferric trichloride hexahydrate (FeCl_3_·6H_2_O), and sodium acetate (NaAc) were purchased from Sinopharm Chemical Reagent Co. Ltd. (Shanghai, China). Ultrapure water obtained from a Millipore water purification system (Milli-Q, Millipore; Bedford, MA, USA).

### Milk samples

Milk samples were purchased from local market and directly used in all the experiments without any pretreatment. In a prior trial of MMIPs-SPE-iEESI-MS, none of FQs such as norfloxacin, enoxacin, and fleroxacin were found in the blank milk samples. A series of standard solutions containing 0–500 *µ*g L^−1^ of FQs including norfloxacin, enoxacin, and fleroxacin were prepared by serial dilution from 0.1 mg mL^−1^ stock solution of FQs in methanol. Note that, to ensure mixed evenly, all the FQs-spiked milk samples were vigorously shaken using a test tube shaker (2800 rpm, Lab Dancer S25, IKA, Germany) before MMIPs-SPE-iEESI-MS analysis.

### SPE based on MMIPs coupled with iEESI-MS analysis

**Figure 5:**
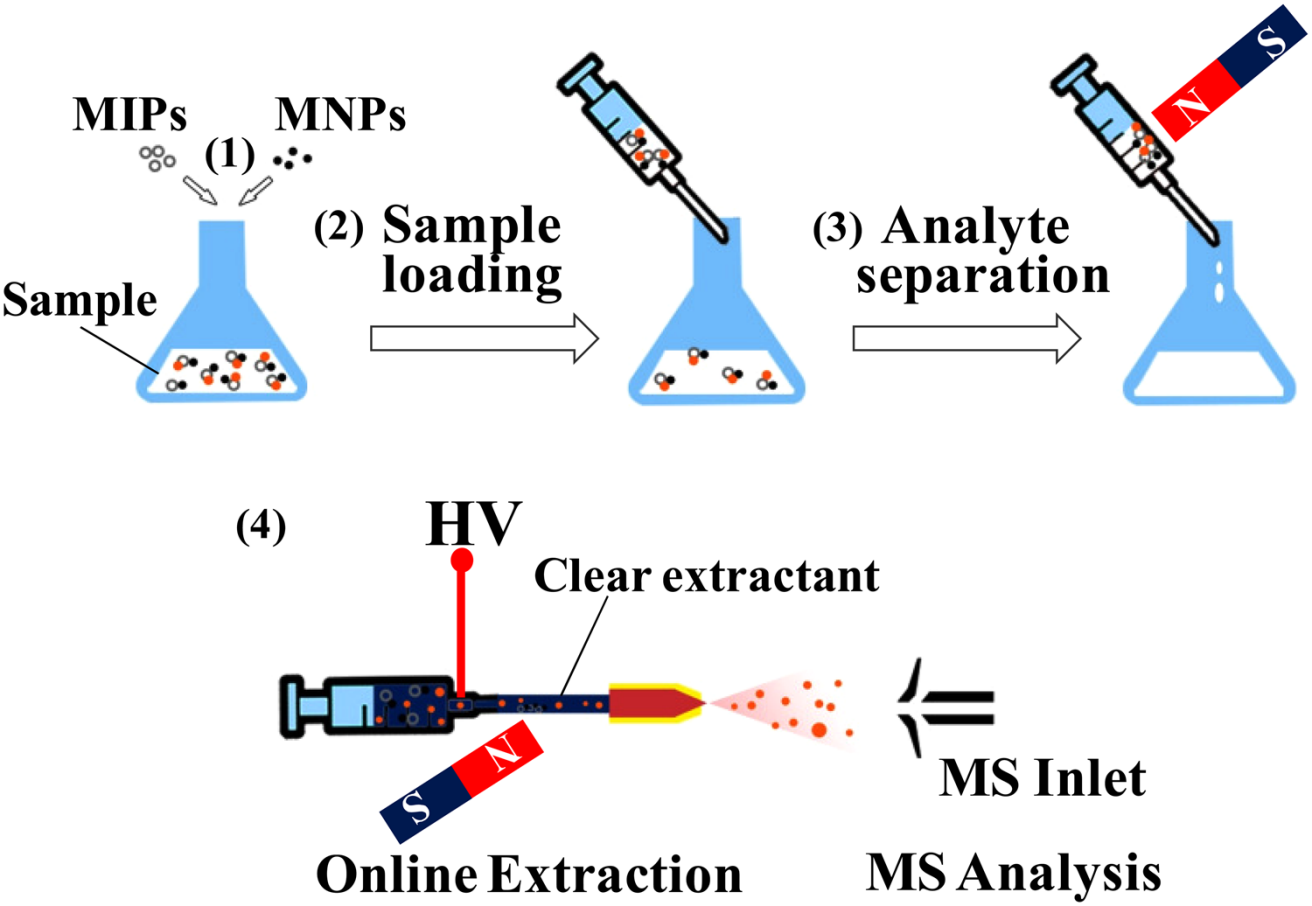
Schematic illustration of MMIPs-SPE-iEESI-MS for quantification of FQs.

The schematic illustration of MMIP-SPE-iEESI-MS for quantification of FQs was shown in Fig. 5. MMIPs were obtained from a simple co-mixing procedure according to the previous literature^46^, *i*.*e*., 2.0 mg Fe_3_O_4_ magnetite nanocomposites (MNPs) and 1.5 mg molecularly imprinted polymers (MIPs) were co-mixed in 1.0 mL methanol by vigorously vortexing for 1 min in a 5-mL glass vial. Then, the methanol was removed from the MMIPs with the assistance of an external magnet and residues of methanol in the MMIPs material volatilized away after about 1 min. The obtained MMIPs were used for the extraction of FQs from milk samples. A 2 mL aliquot of raw milk sample was added into the 5-mL glass vial containing MMIPs material (3.5 mg) and vortexed for 1 min. The suspension mixture was loaded in a 1 mL syringe (Hamilton company, Nevada, USA), and MMIPs captured with FQs were magnetically gathered to the inner wall of the syringe with an external magnet. The milk waste was discharged into a glass beaker. After twice repeats of the MMIPs collection, all the FQs captured MMIPs were gathered on the inner wall of the syringe. To avoid the milk residues interference during the ionization of FQs, the FQs captured MMIPs inside the syringe were washed using 1 mL deionized water, acetonitrile, and 15% acetonitrile in deionized water (v/v), respectively. After loading with 100 *μ*L extraction solution (2% ammonia in methanol, w/w), the syringe was shaken for 20 s to allow the FQs eluted to form a FQs solution which is suitable for electrospray purpose. The FQs solution was pumped through a capillary for ESI at flow rate of 8 *μ*L min^−1^, a strong magnet was placed outside of the capillary to prevent the MMIPs material from reaching to the ESI nozzle. Thus, all the MMIPs material was purposely held by the external magnet and no particles reached to the ion entrance of the mass spectrometer instrument. The MS/MS signal collection duration was 1 min and the average signal intensities of fragment ions were selected from a 30 s window. The average signal intensities of fragment ions of *m/z* 276, *m/z* 277, and *m/z* 326 were selected as analytical response to establish the quantitative method for norfloxacin, enoxacin, and fleroxacin, respectively. It is noted that the lifetime of the MMIPs was about 3 times performances of MMIPs-SPE-iEESI-MS and the performance would decrease significantly over 3 times due to the matrix contamination.

All the experiments were carried out using an Orbitrap Fusion™ Tribrid™ mass spectrometer (Thermo Scientific, San Jose, CA, USA). Mass spectra were collected at the mass range of *m/z* 50–500 under positive ion detection mode. The electrospray solution was pumped at a flow rate of 8 *μ*L min^−1^ using a syringe pump (Harvard Apparatus, Holliston, MA, USA). The ionization voltage was set at +3.0 kV, and the heated LTQ capillary was maintained at 250 °C. The pressure of nitrogen sheath gas was 60 Arb. CID experiments were carried out for MS/MS analysis. During the CID experiments, precursor ions were isolated with a window width of 1.0 Da, and normalized collision energy (NCE) was set to 30–40%. Other parameters were set to instrument default values. Scanning electron microscopes (SEM) and energy dispersive X-ray analysis (EDX) were performed to investigate the morphological, size and elements of MIPs, MNPs, and MMIPs materials by the FIB-SEM instrument (Helios Nanolab 600i from FEI Co., USA). The electron beam and working distance of the instrument were set to 10–20 kV and 4 mm, respectively.

## Electronic supplementary material

Supplementary Information
