# Supplementary material for: Internal Extractive Electrospray Ionization Mass Spectrometry for Quantitative Determination of Fluoroquinolones Captured by Magnetic Molecularly Imprinted Polymers from Raw Milk
Source: Sci Rep. 2017 Nov 7;7:14714. doi: 10.1038/s41598-017-15202-1 (PMC5676746; doi:10.1038/s41598-017-15202-1)
Supplement: Supplementary file 1 — Supplementary Information [file 41598_2017_15202_MOESM1_ESM.doc]

*Supplementary information for*

**Internal Extractive Electrospray Ionization Mass Spectrometry for Quantitative Determination of Fluoroquinolones Captured by Magnetic Molecularly Imprinted Polymers from Raw Milk**

Hua Zhang^1^, Wei Kou^1^, Aisha Bibi^1^, Qiong Jia^1^, Rui Su^1,2^, Huanwen Chen^1,3^, and Keke Huang^1^*,

^1^State Key Laboratory of Inorganic Synthesis and Preparative Chemistry, College of Chemistry, Jilin University, No. 2699 Qianjin Street, Changchun, 130012 P.R. China

^2^Changchun University of Chinese Medicine, No. 1035 Boshuo Road, Changchun, 130117 P.R. China

^3^Jiangxi Key Laboratory for Mass Spectrometry and Instrumentation, East China University of Technology, No. 418 Guanglan Road, Nanchang, 330013 P.R. China

Corresponding author:

Dr. Keke Huang, E-mail: kkhuang@jlu.edu.cn, Tel: (+86)431-8516-8624. Fax: (+86)431-8516-8661.

**1. Preparation of Fe_3_O_4_ magnetite nanocomposites (MNPs)**

Fe_3_O_4_ magnetite nanocomposites (MNPs) were prepared according to previous studies.^1, 2^ Briefly, FeCl_3_·6H_2_O (2.0 mg) was dissolved in ethylene glycol (40.0 mL), and then NaAc (6.0 g) and ethylene diamine (20.0 mL) were added into the solution. After vigorous vortexing at room temperature for 30 min (800 rpm), the homogenate was sealed in a Teflon-lined stainless-steel autoclave (100 mL). The autoclave was heated to 200 °C and maintained for 8 h. The obtained Fe_3_O_4_ magnetite nanocomposites product was cooled at room temperature and magnetically collected with the assistance of an external magnet. The solids product was washed with water/ethanol (v/v, 1/1) and dried in vacuum at 60 °C for 6 h to obtain MNPs.

**2 The determination of** **fluoroquinolones (FQs) in milk by LC-MS/MS**

The LC-MS/MS experiment was carried out following a standard operation procedure recommended on *National Standard of China (GB/T* *22985-2008).*

2.1 Extraction

2.00 g milk was weighted carefully and loaded in a 50 mL plastic centrifuge tube with 10 mL acetonitrile added inside. Then the mixture was oscillated and extracted using a vortex oscillator for 1 min. The homogenate was centrifuged at a rate of 5000 rpm for 5 min and the obtained supernatant was filtered into a heart-shaped bottle. Then, 5 mL of phosphate buffer solution (Na_2_HPO_4_-KH_2_PO_4_, 0.05 mol/L, pH 3.0) and 10 ml of acetonitrile were added to the residue, and repeated the above steps to separate the supernatant again. Merge all the supernatants, followed by complete evaporation of the supernatant to concentrate the analytes using rotary evaporator (50 °C). After all the acetonitrile vaporized out, 5 mL of phosphate buffer solution was added inside to dissolve the analytes.

2.2 Purification

The sample solution was purified using an Oasis HLB solid phase extraction column (Waters Corporation, MA, USA). Briefly, analytes solution prepared above was transferred to go through the HLB solid phase extraction column at a flow rate of 1 mL/min. After the sample solution completely went through, the column was washed with 4 mL water and 4 mL 25% methanol in water solution (v/v), respectively, and vacuum drained the column. Then, 4 mL elution solution (2% ammonia in methanol, w/w) was employed to elute the analytes and the eluent was collected using a 10 mL centrifuge tube. The obtained eluate was dried to about 0.2 mL using nitrogen gas at 50 °C, then stop concentration. The concentrated sample was diluted to 1 mL with a methanol-formic acid solution (15/85, v/v) and centrifuged at a rate of 5000 rpm for 5min. The supernatant was filtered using an organic filter (membrane pore size of 0.22 *µ*m) to obtained sample solution suitable for LC-MS/MS analysis.

2.3 LC-MS/MS analysis

The HPLC instrument used in this study was from Agilent Technologies (Palo Alto, CA, USA) series 1200 capillary HPLC, which coupled to an Applied Biosystems (Waltham, MA, USA) API 4000™ triple quadrupole mass spectrometer through an electrospray ionization source.

*Parameters of HPLC:* Chromatographic separation was accomplished using a 150 mm long by 2.1 mm i.d. microcolumn packed with 3.5 *µ*m Agilent ZORBAX SB-C_18_ stationary phase operated at 30 °C. The mobile phase composition used was 0.1% formic acid in water (mobile phase A) and acetonitrile (mobile phase B) delivered at a flow rate of 250 *μ*L/min. Linear gradient procedure was as follows (1) 85% A decreased to 10% A over 0–4 min, (2) maintained 10% A over 4–12 min, (3) 10% A increased to 85% A over 12–12.5 min, and (4) kept 85% A over 12.5–18 min. The column heater was set to 30 °C and injection volume was set to 20 *μ*L.

*Parameters of MS analysis:* The mass spectrometer was used with the regular ESI interface and calibrated prior to experiments. Multiple reaction monitoring (MRM) mode with positive electrospray ionization (ESI+) was performed for the detection of FQs. Ionization voltage was +5.5 kV, the temperature of the ion source was 600 °C, curtain gas (CUR) was 40 psi, nitrogen sheath gas was 45 pis, auxiliary heating gas was 40 psi, and collision gas was 9 psi. Collision energy was set to 25 V–35 V and declustering potential was kept at 70 V.

**Figure S1. Elemental analysis of the MIPs (a), Fe_3_O_4_ MNPs (b), and MMIPs (c).**

**Figure S2. Comparison of the performance of Fe_3_O_4_ MNP and MMIP materials.** Abbreviations: NOR, norfloxacin; FLE, fleroxacin; ENO, enoxacin.

**Tab. S1 Analytical results for MMIPs-SPE-iEESI-MS/MS analysis of blank milk and norfloxacin spiked milk samples.**

**Tab. S2 Analytical results for MMIPs-SPE-iEESI-MS/MS analysis of blank milk and enoxacin spiked milk samples.**

**Tab. S3 Analytical results for MMIPs-SPE-iEESI-MS/MS analysis of blank milk and fleroxacin spiked milk samples.**
